# Characterization and Evaluation of a Commercial WLAN System for Human Provocation Studies

**DOI:** 10.1155/2015/289152

**Published:** 2015-06-09

**Authors:** Norbert Zentai, Serena Fiocchi, Marta Parazzini, Attila Trunk, Péter Juhász, Paolo Ravazzani, István Hernádi, György Thuróczy

**Affiliations:** ^1^Department of Experimental Neurobiology, University of Pécs, 6 Ifjúság Útca, Pécs 7624, Hungary; ^2^Consiglio Nazionale delle Ricerche (CNR), Istituto di Elettronica e di Ingegneria dell'Informazione e delle Telecomunicazioni (IEIIT), Piazza Leonardo da Vinci 32, 20133 Milano, Italy; ^3^Szentagothai Research Center, University of Pécs, Ifjúság Útca 20, Pècs 7624, Hungary; ^4^National Institute for Radiobiology and Radiohygiene, Anna Útca 5, Budapest 1221, Hungary

## Abstract

This work evaluates the complex exposure characteristics of Wireless Local Area Network (WLAN) technology and describes the design of a WLAN exposure system built using commercially available modular parts for the study of possible biological health effects due to WLAN exposure in a controlled environment. The system consisted of an access point and a client unit (CU) with router board cards types R52 and R52n with 18 dBm and 25 dBm peak power, respectively. Free space radiofrequency field (RF) measurements were performed with a field meter at a distance of 40 cm from the CU in order to evaluate the RF exposure at several signal configurations of the exposure system. Finally, the specific absorption rate (SAR) generated by the CU was estimated computationally in the head of two human models. Results suggest that exposure to RF fields of WLAN systems strongly depends on the sets of the router configuration: the stability of the exposure was more constant and reliable when both antennas were active and vertically positioned, with best signal quality obtained with the R52n router board at channel 9, in UDP mode. The maximum levels of peak SAR were far away from the limits of international guidelines with peak levels found over the skin.

## 1. Introduction

Wireless Local Area Networks (WLANs) using Wireless Fidelity (Wi-Fi) technology are commonly used in various environments such as office buildings, schools, and homes [1]. Dedicated devices, called access points (APs), are strategically placed in the environment to provide signals for Wi-Fi communication service. Due to the broad diffusion of WLAN systems, users are exposed to the electromagnetic fields (EMFs) irradiated by WLAN networks during the long periods of operation. Moreover, since in WLAN systems the antenna(s) of the client device are usually close to the body of the user, relatively high energy may be absorbed in the body as indicated by increased specific absorption rate (SAR), which is the metric used to assess the absorption of the signal in the living body and refers to the compliance with safety standards and guidelines (see exposure limits guidelines [2]).

Exposure to radiofrequencies (RF) emitted by WLAN systems has been raising a serious public concern about possible human health effects. Although the issue of possible adverse health effects of RF energy emitted in the Ultra High Frequency range (UHF, 300 MHz–3 GHz) has been addressed for some technologies such as mobile phones [3–7] or Wi-Fi [8–17] and partially addressed for others such as Bluetooth, Digital Enhanced Cordless Telecommunications (DECT), and Radio Frequency Identification (RFID) [18–21], these concerns open new avenues for further investigations on the possible biological effects of exposure of specific WLAN signals.

In the bioelectromagnetics research area, it is well known that no study of the effects can be considered as reliable without completely characterized exposure systems used for exposing the target agents, such as cells, tissues, whole body animal subjects, or human volunteers [22]. However, up till now only a few papers have investigated the best laboratory practice to be applied in implementing an exposure system specifically designed for Wi-Fi experiments [23–29], and available literature data are mainly devoted to test compliance with safety standards and guidelines not available for biological studies.

Therefore, the present study aims to evaluate the complex RF exposure characteristics of WLAN technology and describe the design and evaluation of a specific WLAN exposure system using Wi-Fi modulation made suitable for human studies aiming at investigating potential biological effects of Wi-Fi exposure, particularly on human brain function.

The performance of the newly developed exposure system was characterized in terms of wireless cards used, directional characteristics of antennas, power stability as a function of channels, data rate, transmission protocol, and output power. A realistic usage scenario for investigations on possible effects of WLAN exposure on human brain function was also assessed by testing the system to the boundaries of the utilization rate. The assessment of the level of exposure was presented in terms of SAR as performed by computational electromagnetics techniques on two realistic human whole body voxel models.

## 2. Experimental Section

Several Wi-Fi standards, characterized by different frequency bands of the radio spectrum, are currently available on the market. All marketed Wi-Fi products are based on the IEEE Standard 802.11 and comply with several dedicated protocols. Usually Wi-Fi systems are based on the IEEE Standards 802.11b and 802.11g and operate in the 2.4 GHz frequency band. According to the various local legislations and regulations, Wi-Fi devices which are designed for private (home) use should emit low power (less than 20 dBm or 100 mW) and should work in a frequency band also used by other communication devices (such as cordless phones). Wi-Fi devices based on the IEEE Standard 802.11a operate in the frequency band of 5.8 GHz and are suitable to be used in public environment. The IEEE Standard 802.11n works in both frequency bands of 2.4 and 5.8 GHz. To sum up, so far, the most commonly used technologies are 802.11b and 802.11g (2.4 GHz, maximum output power 100 mW) and the 802.11a (5.8 GHz, maximum output power 1 W).

### 2.1. Wi-Fi Exposure System

**Figure 1:**
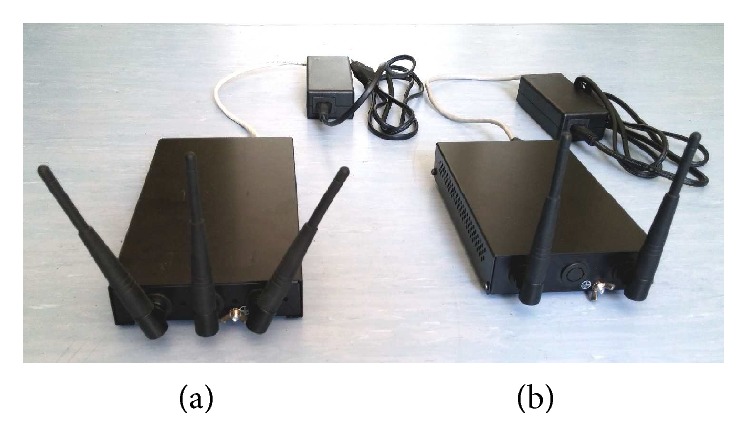
Picture of the complete WLAN exposure system. (a) The access point (AP) with the three Swivel omnidirectional antennas. (b) The client unit (CU) with the two Swivel omnidirectional antennas.

In the present study, a WLAN exposure system was constructed from commercially available parts that were all produced by MikroTik (Riga, Latvia) (Figure 1).

During the structuring of the WLAN exposure system for the purpose of biological experiments, two types of mPCI (mini Peripheral Component Interconnect) router board cards, one 802.11 a/b/g (type R52) and one 802.11a/b/g/n capable (type R52n), were evaluated nominally with 20−80 mW (13−19 dBm) and 20−200 mW (13−23 dBm) peak power, respectively. Both the R52 and R52n router board cards were operated only on b/g transfer mode and never on a- or n-transfer mode. Both cards were capable of operating in the 2.4 GHz or 5.8 GHz frequency bands and both had U.FL antenna connectors.

The WLAN system consisted of an access point (AP) and a client unit (CU). The AP consisted of a commercially available router motherboard MikroTik RB433AH with 680 MHz mipsbe processor, 128 MB RAM, with three mPCI connectors and three Ethernet ports. The system ran on RouterOs Level5 v4.10 with AP support. The motherboard was placed in a custom-built aluminum case RB433U, with three AC/SWI Swivel holes for three AC/SWI 2 dBi Swivel omnidirectional antennas with an U.FL antenna connector, and operated with 24 V/1.6 A Power Over Ethernet (POE) power supply via CAT5 UTP cable.

The CU consisted of a router motherboard MikroTik RB411AH with a similar 680 MHz mipsbe processor, 64 MB RAM, with one mPCI and one LAN port, and ran with RouterOs Level4 v4.10 with AP support, in an indoor case RB411, with three AC/SWI Swivel holes for two AC/SWI 2 dBi Swivel omnidirectional antennas with U.FL antenna connector, operating with a 24 V/1.6 A POE power supply via CAT5 UTP cable. The AP and the CU were both controlled by a personal computer (PC) applying WinBox v2.2.18 software (MikroTik Co, Riga, Latvia) running under MS Windows operating system (Microsoft Co, Redmond, US).

### 2.2. Characterization of Antenna Radiation Pattern

The two CU antennas were characterized by experimental estimation of the antenna radiation. Both antennas were kept active and in upright (vertical) position. Results were compared with the computational radiation pattern obtained using the commercial platform SEMCAD X (Schmid & Partner Engineering AG, Zurich, Switzerland). The exposure unit was modelled according to the realistic dimensions of the unit (aluminum case size: 180 × 112 × 31 mm, dipole antennas length: 113 mm, and distance between the two antennas: 54 mm). The model was fed with a 2.4 GHz signal pattern with an input power of 20 dBm (100 mW).

**Figure 2:**
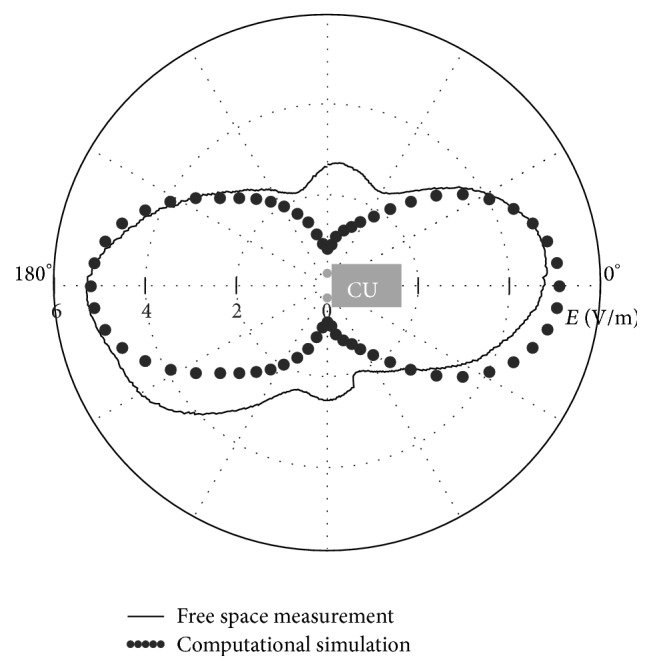
Typical radiation pattern of the WLAN exposure system performed by free space measurement at 20 dBm output power at *d* = 40 cm with both antennas active and in upright position (continuous line) and results of computational simulation of the same antenna arrangement (dotted line).

Figure 2 shows the electric field magnitude as measured from 40 cm distance with both antennas being active and kept in an upright position and also the results of a computational simulation using the same arrangement. An almost equivalent electric field magnitude was obtained comparing measurements and simulations (5.37 V/m, with an uncertainty of less than 2%).

### 2.3. Free-Field Measurement Setup

**Figure 3:**
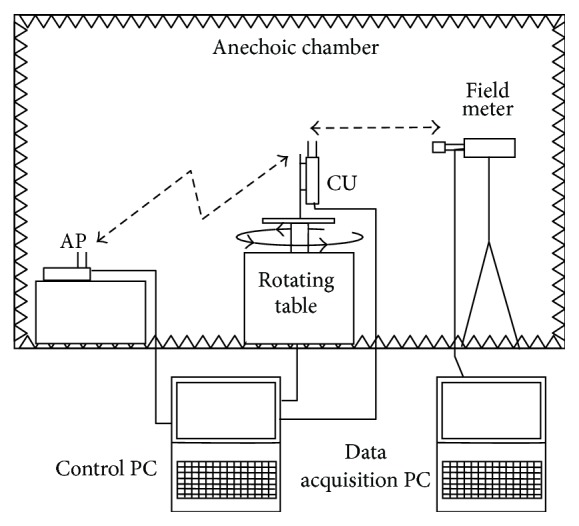
Schematic diagram of the experimental setup for free-field measurement of the WLAN system.

For the free-field measurements both the server (i.e., AP) and CU were placed in an anechoic chamber and the RF field near the client was measured with a Narda PMM 8053 field meter (Narda Safety Test Solutions, Savona, Italy) with a wide-band E-field probe. The distance between the CU antennas and the field meter probe was 40 cm, which is aimed at modelling the typical distance between the user and the personal computer (e.g., notebook) in which Wi-Fi antennas are often mounted in the frame of the screen.  Figure 3 shows an overview of the free-field measurement setup. The two PC that controlled the AP and CU units via Ethernet cables and were used for data acquisition from the field meter were placed outside the anechoic chamber in order not to perturb the electric field inside the chamber. To measure the directional characteristics the CU was fixed on the top of a programmable rotating platform with a rotational axis placed in the middle between the two antennas, while the field meter remained stationary (Figure 3).

Several WLAN signal configurations were tested. Directional characteristics with one antenna active versus both antennas active, as well as both antennas folded versus both antennas operating in upright position, were compared. To ensure the stability of the RF output, a bandwidth test was initiated over the wireless interface from the CU through the WinBox control software for the duration of one hour. The effects of various settings were then investigated: the electric field was measured with different protocols (Transmission Control Protocol, TCP, versus User Datagram Protocol, UDP), channels (1–13), modes (802.11b versus 802.11bg versus 802.11bn), and data rates (1/5.5/11 Mbps for IEEE Standards “b/g” and 6/24/54 Mbps for “g”).

To collect the packet length and interval statistics necessary to calculate duty cycles of various modes, a small antenna (AR006-WO1, Wellshow Technology Co Ltd., Taiwan) was placed near the CU. The signal from that antenna was rectified with a microwave detector (NardaType-503, Hauppauge, NY, USA) and the resulting DC signal was digitized with a high performance 16-bit A/D converter (CED Micro1401, Cambridge, UK) at a sampling rate of 100 kHz. First, a bandwidth test was initiated from the CU to test modes with the highest utilization rates possible; then a wireless data connection was set up between the AP and the CU. A file download was also performed from a local file server to test a more realistic usage scenario. Both download and upload scenarios were tested. When the client was downloading a file, it only sent the acknowledgement (ACK) packages associated with TCP mode. These measurements were repeated with the data rates restricted to different, fixed values (1/5.5/11 Mbps for 802.11b/g and 6/24/54 Mbps for 802.11g).

### 2.4. SAR Computational Estimation

The human exposure to the RF energy emitted by the Wi-Fi exposure system was also performed by computational modeling of SAR. In this study, the commercial simulation platform SEMCAD X was used to estimate the SAR levels in realistic anatomical human models. The results were presented in terms of 10 g averaged SAR over tissues (peak SAR_10 g_), normalized by considering a nominal input power of 20 dBm (100 mW) and a duty cycle of 100% of the Wi-Fi signal. Those SAR levels were computed following the recommendations of the IEEE Standard C95.3 [30] and where the mass of the tissue is <10 g following the IEEE Standard 1528 [31].

**Figure 4:**
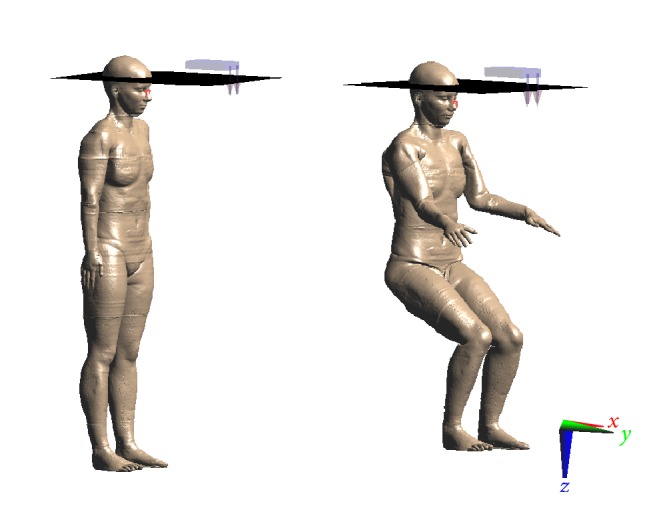
The positions for computational modeling of SAR distribution for the female human voxel model “Ella.”

The CU was modelled as based on the real geometry and the physical characteristics of the device: the case was modelled as an aluminum box with dimensions 180 × 112 × 31 mm. The two antennas were taken as active and operating in upright (vertical) position with 113 mm of length, with a distance of 54 mm between the antennas, and taken as dipoles to obtain a resonance frequency of 2.54 GHz. Two whole body, realistic human models were used, both from the Virtual Family (“Ella,” female, 26 years old, 1.63 m height, 58 kg weight, BMI: 22.0 kg/m^2^ and “Duke,” male, 34 years old, 1.77 m height, 72.4 Kg, BMI: 23.1 kg/m^2^), available for research purposes [32]. The models were used in standing and sitting positions (Figure 4), the latter obtained with the “Poser” tool available within the simulation platform SEMCAD X. The model design was based on high-resolution magnetic resonance (MR) images of healthy volunteers, segmented in a voxel-based format at a resolution of 1 mm. Thus, the human models allowed distinguishing up to 77 tissues. The dielectric properties of those tissues were assigned according to the classical parametric model described by Gabriel et al. (1996) [33]. The modelled CU was placed in such a way that the middle point between the two horn antennas was in line and 40 cm away from the intersection of the frontal bone and two nasal bones (i.e., the “nasion”) of the model. A nonuniform mesh with a maximum spatial step of 3 mm (in free space) restricted to 1 mm over the anatomical model and to 0.2 mm over the CU was adopted to discretize the computational domain. The cited positions of the human models were chosen to mimic a realistic notebook use, and as such (also shown in some previous studies [34, 35]) one may expect a considerable rise in whole body SAR and even more in peak SAR when the posture is changed from standing position.

## 3. Results and Discussion

### 3.1. Directional Characteristics

First, the radiation pattern of the WLAN exposure system was recorded in free space for three different antenna positions: (i) one antenna active and positioned horizontally and the other inactive and positioned vertically (*E*max = 4.09 V/m); (ii) one antenna active and positioned vertically and the other inactive and positioned horizontally (*E*max = 3.14 V/m); (iii) both antennas active and positioned vertically (*E*max = 5.29 V/m). As described above (Section 2.2), the latter configuration (iii) being the one that produces the highest electric field was also simulated and chosen as golden standard for the characterization of the WLAN system.

### 3.2. Electric Field Strength and Signal Characteristics of WLAN System

**Figure 5:**
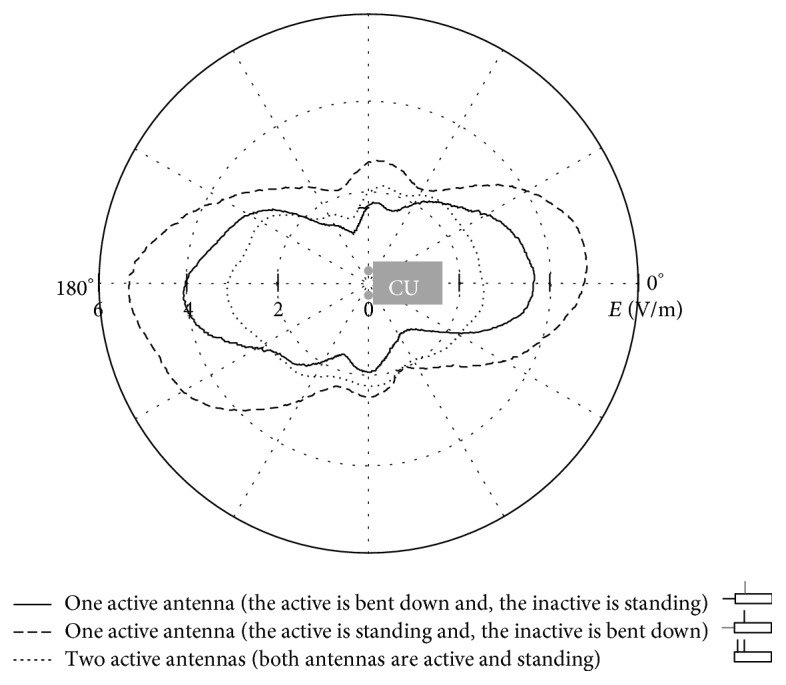
Radiation pattern of the WLAN exposure system performed by free space measurement at 20 dBm output power at *d* = 40 cm with the antennas taken in the three different configurations listed in the legend.

To assess the radiation features of the system, the electric field strength (in terms of electric field magnitude *E*) was measured as a function of different parameters. The results are described from Figure 5 to Figure 11.

**Figure 6:**
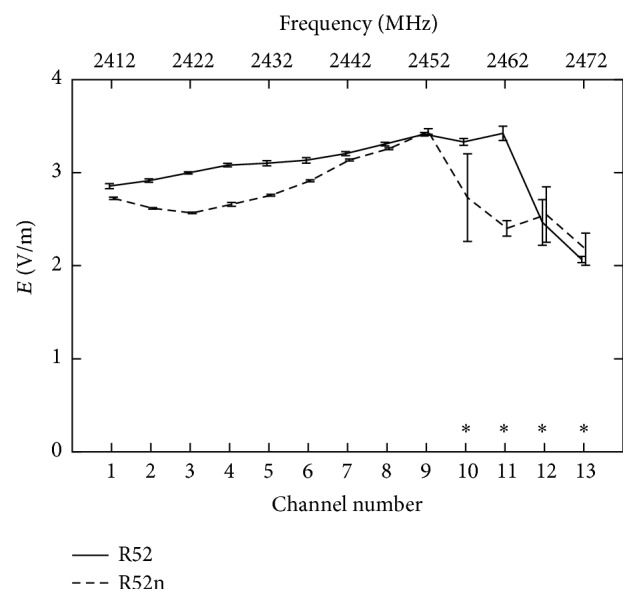
Dependence of measured electric field at *d* = 40 cm on the frequency (channel number) for R52 (solid line) and R52n (dashed line) router board cards under test with UDP mode and one active antenna in vertical position. Asterisks mark channels where performance breakdown occurs (meaning that the card was unable to send data at the full data rate). Error bars represent mean ± standard deviation.

Figure 6 shows the behavior of the electric field strength as a function of the channel and the corresponding frequency, for both R52 and R52n router board cards. Notice that both the R52 and R52n router board cards were utilized only on b/g transfer modes, never on a-mode or n-mode. The measurements were originally performed with a default system setting with both antennas upright (standing) and one of the antennas active and transmitting in UDP mode. In this setting using channels 10 to 13 and at frequencies higher than 2.452 GHz the signal quality and strength declined at full data rate indicating the breakdown of the performance in sending packets. Therefore, we avoided these channels for designing data transmission protocols for the planned WLAN exposure system. On the contrary, channel 9 guaranteed the maximum level of *E* for both router board cards, and therefore, channel 9 was considered the most suitable to be used in studies on possible biological effects of Wi-Fi exposure.

**Figure 7:**
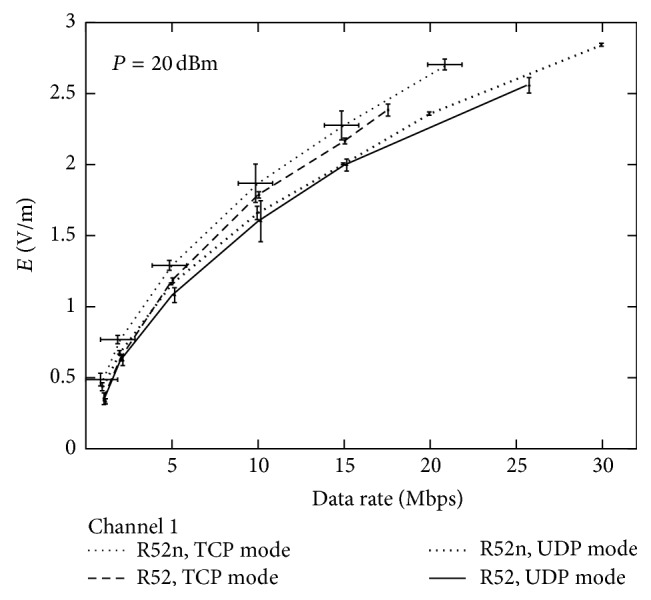
Measured electric field strength as a function of nominal data rate with UDP or TCP data transfer mode (as set by the router configuration utility) with using a R52 or R52n router board card. Only data for channel 1 are shown here to allow comparison between the different modes. A fixed power level of 20 dBm was used.

In order to further describe the WLAN system, performance measures on channel 1 were tested (Figure 7) in terms of electric field strength as a function of the nominal data rate of the different router board cards (R52 and R52n), using two different protocols (TCP and UDP). The RF power was fixed at 20 dBm. The best performances were obtained by using TCP mode and R52n router board card, which, at any data rate, achieved higher power than in the UDP mode and the R52 card.

**Figure 8:**
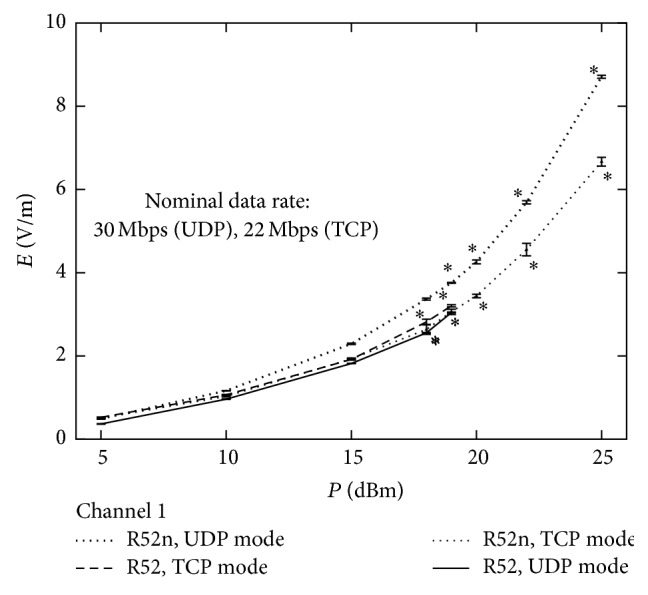
Measured electric field strength as a function of nominal output power with UDP or TCP data transfer mode (as set by the router configuration utility) with using R52 or R52n router board card. Data rate was left unconstrained for this test. Asterisks mark the cases where the router board was unable to generate the maximum data rate (30 Mbps). Only data for channel 1 is shown here to allow comparison between the different modes.

The electric field strength generated by the two wireless cards with the two different transmission protocols for channel 1 was evaluated as a function of the nominal output power as set by the router configuration utility (in WinBox software) (Figure 8).

**Figure 9:**
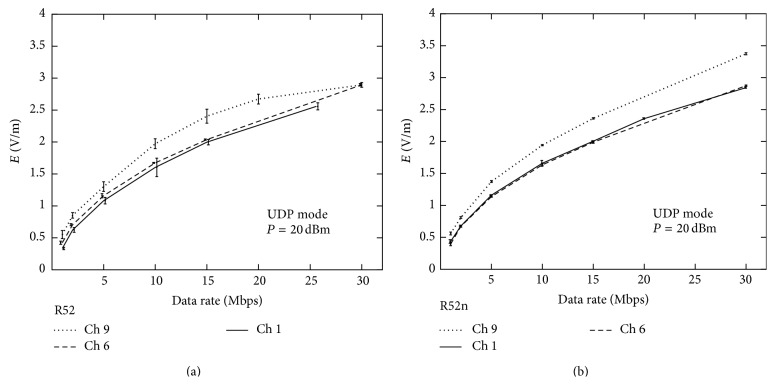
Comparison of data from channels 1, 6, and 9 in UDP mode with using (a) R52 and (b) R52n router board card.

Data rate was left unconstrained, but the cases of the test, in which the card was unable to transmit data at the maximum frequency, were marked with asterisks in the figure. As one can notice system breakdown often occurred for power over 18 dBm (70 mW). Results show that the UDP mode appeared slightly more appropriate compared to TCP mode in generating higher power for the R52n router board card and less appropriate for the R52 card. The two router board cards were also tested against the data rate, considering the three default channels that are most commonly used in commercial routers channels: channel 1, channel 6, and channel 9 (Figure 9). The tests were performed fixing the power (20 dBm or 100 mW) in UDP mode. Channel 9 was found to emit larger electric field than channel 1 and channel 6, for both R52 and R52n router board cards.

**Figure 10:**
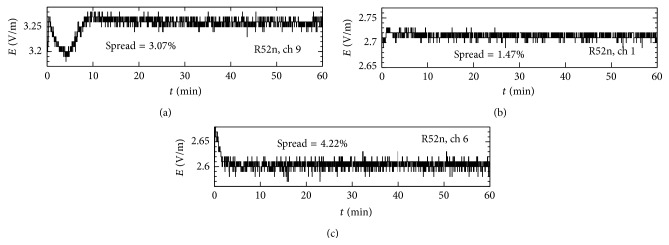
The stability of the output power over a one-hour continuous testing period with the R52n router board card on (a) channel 9, (b) channel 1, and (c) channel 6.

Moreover, the stability of the output power on the same three channels was also checked over one-hour period with both cards, finding that the fluctuation of the output power is almost negligible with router board card R52n, worse with card R52 on channel 9 (spread = 30.78%).  Figure 10 indicates the results of the test obtained with the R52n router board card.

**Figure 11:**
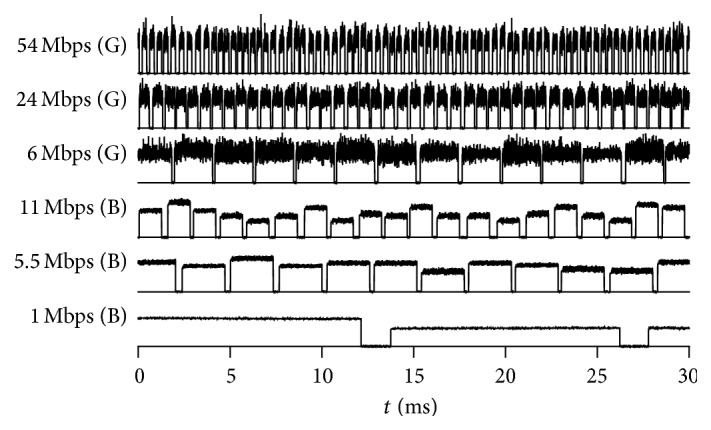
Example waveforms generated by the WLAN exposure system. The data rate and the connection mode between the AP and the CU were locked, and then a bandwidth test was initiated from the CU (1500 bytes/frame, UDP mode). (G) and (B) represent data transfer modes 802.11g and 802.11b, respectively.

The waveforms of the RF radiation emitted by the WLAN system were also tested in order to describe the modulation of the exposure as modulation itself may have a key role in the biological response to the RF exposure. The data rate and the connection mode between the AP and the CU were locked and then a bandwidth test was initiated from the CU (1500 bytes/frame, UDP mode). The waveforms were captured by means of an antenna located near the CU. The signals from the receiver antenna were rectified by a detector diode and sampled by a 16-bit A/D converter (CED Micro1401, Cambridge, UK) on 100 kHz sampling rate. Figure 11 shows example waveforms generated by the WLAN exposure system on a common time scale.

One final remark about these measurements is to take into consideration that our setup was tested only under these conditions, with these parts and parameters. We recommend for potential later other experiments to use exactly the same brand and types of parts and parameters of setting because the system characteristics are not tested and thus cannot be guaranteed at other conditions.

### 3.3. Computational SAR Calculation

**Figure 12:**
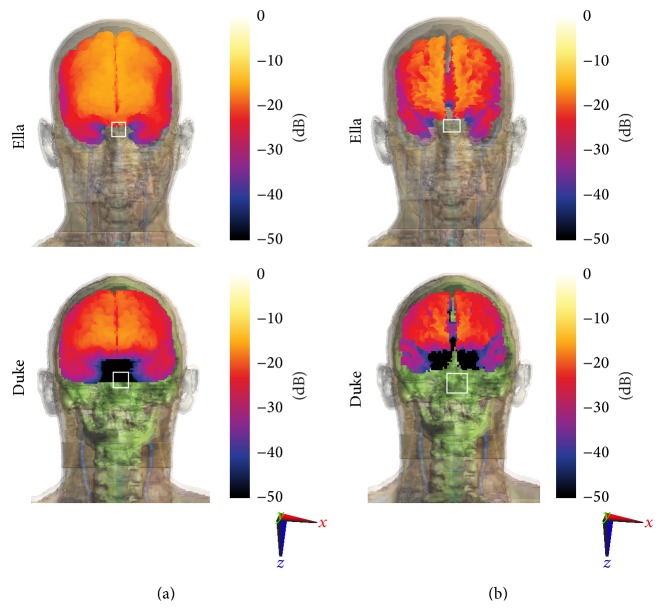
SAR_10 g_ distributions over (a) grey matter and (b) white matter of “Ella” (1st row) and “Duke” (2nd row). Scaling bar is normalized to the peak SAR_10 g_ found over each model (i.e., 22.77 mW/kg and 10.37 mW/kg for Ella and Duke, resp.). White squares indicate the maxima peak SAR_10 g_ locations.

Figure 12 shows an example of exposure assessment performed by computational electromagnetics on the heads of the two human models, considering as source the CU being fed at 20 dBm (100 mW and 100% duty cycle) at 2.45 GHz and a distance between head and CU antennas of 40 cm, tailored for an experimental study on human volunteers. In light of some contrasting results found by studies on the effects of acute RF-EMF exposure and specifically those of Wi-Fi exposure on higher-order cognitive functions as well as on brain physiology (see, e.g., [36–45]), we believe that it would be interesting to assess the power absorption in specific brain structures involved in performance in cognitive tasks. The target tissues for exposure were assumed to be the forehead skin and the underlying brain tissue such as the cortex (grey matter) and axonal pathways (forebrain white matter). White squares in the figure indicate the maxima peak SAR_10 g_ locations. The results refer to the sitting positions: however, given the same head-CU distances taken for the two simulated positions, they are almost equivalent (differences below 5%).

The peak SAR values obtained in the different tissues are shown in Table 1, for both the considered models. As expected, the target tissues closer to the Wi-Fi source (forehead skin, grey matter, and white matter) are showing higher SAR values, with respect to the cerebellum (positioned on the back of the head with respect to the source and with respect to the other tissues in a deeper position inside the head). The large variability between the maxima peak SAR levels found in the two models is due to the large differences in morphology and thickness of the head tissues of Duke and Ella models, which, as shown in several studies [46–48], is one of the main contributions affecting the induced electric field and hence SAR distributions. Both types of exposure assessment resulted in SAR values largely lower than the maximum values suggested by the ICNIRP guidelines [2] (i.e., 2 W/kg for the peak SAR_10 g_). As last remark in the interpretation of these results, one should bear in mind the different sources of uncertainty intrinsic to the computational simulation which affect the estimated SAR levels. An extensive discussion together with the analysis of the total expanded uncertainty of the peak SAR_10 g_ due to the exposure to EMF in the GHz band is discussed in [20, 49]. 

## 4. Conclusions

This study aimed to develop a WLAN communication system and describe it with a heuristic characterization method suitable for (bioelectromagnetic) measurements in living tissues. WLAN systems usually apply a complex and sophisticated “signal cocktail” modulation of RF radiation. This fact must be taken into consideration in any exposure assessment and application for experimental studies.

Going more deeply into the results, the computational exposure assessment of SAR of the present WLAN system provides seminal data in human body regarding the spatial distribution of RF power absorption in the human head. This is of crucial importance in (1) safety assessment of such exposure systems and for (2) the interpretation of the results of the biological investigations. The highest levels of SAR_10 g_ were found in the tissues closest to the source. On the contrary, on the contralateral side from the source and in deeper tissues SAR_10 g_ levels showed a decrease of more than 26 dB. The results of the present study also indicate that the computationally estimated levels of SAR were well below the ICNIRP limits [2] with the maximum levels found over the skin and in tissues with highest water content, where the absorption of the WLAN signal was relatively high. Indeed, as the frequency increases, the penetration depth decreases, which means that most of the circulating RF energy was absorbed in the skin of the body. Actually, the skin is the body's first line of defence. It protects the body against any external aggressors such as bacteria, fungi, and several physical agents (e.g., UV and temperature). The skin also has an important role in the immune system of the human body. The new emerging wireless technologies (such as UMTS, DECT, LTE, Wi-Fi, and body-worn emerging devices) use higher frequencies than those of GSM 900 MHz systems; therefore the skin, being the most exposed tissue for WLAN system, may have an important role in the possible (chronic) health effects which may arise from extensive RF irradiation.

Results also reflect the general need and importance of prior verification of any custom-built Wi-Fi exposure system with rigorous and heuristic methodology in terms of exposure parameters to reach the best combination settings and assurance for replicability in terms of channels, operation modes, and types of protocol (and interface cards). Based on the present data, the currently investigated WLAN exposure system that consisted of commercially available parts, we conclude that the exposure is more reliable and stable when both antennas are active. The best signal quality and strength were observed using channel 9, UDP mode, and R52n router board and mPCI cards. This setting altogether resulted in an almost negligible output power fluctuation.

Taken together, the present WLAN exposure system, originally built from commercially available parts and tested for suitability for experimentation functions as a reliable and well-controllable signal source for biological studies, aims to test effects of EMFs emitted by Wi-Fi technology in a heuristically controlled, realistic environment.

## Figures and Tables

**Table 1:** Peak SAR averaged over 10 g of contiguous tissue calculated by computational dosimetry over some cerebral tissues of the two anatomical adult models (“Ella” and “Duke”). The levels were obtained by considering a nominal input power of 20 dBm (100 mW) and a duty cycle of 100% of the Wi-Fi signal.

Peak SAR_10 g_ [mW/kg]		
Tissue	Ella	Duke
Forehead skin	22.77	10.37
Grey matter	2.70	1.60
White matter	1.16	0.73
Cerebellum	0.06	0.02
Hippocampus	0.04	<0.01
Hypothalamus	0.01	<0.01
Midbrain	0.01	<0.01
Pons	<0.01	<0.01
Thalamus	0.02	<0.01
